# Development of Communication Behaviour: Receiver Ontogeny in Túngara Frogs and a Prospectus for a Behavioural Evolutionary Development

**DOI:** 10.1100/2012/680632

**Published:** 2012-05-02

**Authors:** Alexander T. Baugh, Kim L. Hoke, Michael J. Ryan

**Affiliations:** ^1^Section of Integrative Biology, The University of Texas at Austin, Austin, TX 78712, USA; ^2^Department of Migration and Immunoecology, Max Planck Institute for Ornithology, 78315 Radolfzell, Germany; ^3^Department of Biology, Colorado State University, Fort Collins, CO 80523, USA; ^4^Smithsonian Tropical Research Institute, P.O. Box 0843-03092, Balboa Ancón, Panama

## Abstract

Most studies addressing the development of animal communication have focused on signal production rather than receiver decoding, and similar emphasis has been given to learning over nonlearning. But receivers are an integral part of a communication network, and nonlearned mechanisms appear to be more ubiquitous than learned ones in the communication systems of most animals. Here we review the results of recent experiments and outline future directions for integrative studies on the development of a primarily nonlearned behaviour—recognition of communication signals during ontogeny in a tropical frog. The results suggest that antecedents to adult behaviours might be a common feature of developing organisms. Given the essential role that acoustic communication serves in reproduction for many organisms and that receivers can exert strong influence on the evolution of signals, understanding the evolutionary developmental basis of mate recognition will provide new insights into the evolution of communication systems.

## 1. Introduction

Seminal discoveries in the study of embryonic patterning such as the deep homology of *Hox* gene clusters in animal evolution spurred the emergence of the evo-devo as a discipline [1]. As a field evo-devo integrates the role of development into our understanding of evolution with the potential to greatly expand the framework of the modern synthesis. While most studies in evo-devo explore embryonic development, it is a critical next step to extend this conceptual framework to postembryonic stages of development—the period in an organism's life when not only development does not cease, but also behaviours arise and differentiate. And while in many organisms the onset and differentiation of behaviour during this time are strongly influenced by social experience [2] or self-feedback [3], there are also many examples in which behaviours emerge in a largely experience-independent manner (e.g., migration [4], predator recognition [5], and mating signals [6]). In this regard, one particularly promising area of study for behavioural evo-devo is communication. Communication signals subserve vital social functions and yet the production and recognition of such signals are, for many species, nonlearned [7–10]. Although there are volumes of research exploring the behavioural evolution or behavioural development of communication, few, if any, studies integrate these usually independent research streams. While there are many intriguing systems that could be pursued from this integrative perspective [11, 12], we focus here on studies and prospects in the area of communication in a tropical frog because we believe this emphasis and system can provide some general principles relevant to the development and evolution of behaviour.

## 2. What Are the Key Features for the Study of the Development of Communication Behaviour?

Study systems that have provided models of behavioural development share several key features [13, 14]. First, the behavioural traits and their precursors must be reliably identifiable, and for communication behaviour, they are ideally stimulus elicited. Second, developmental trajectories must be long enough in duration to permit multiple repeated measures, preferably using a within-subject design to control for what are sometimes large between-subject variances developmentally (e.g., rates of physiological or neural development), but short enough to enable time-efficient longitudinal studies. Third, the behaviours under study should be ethologically informed and relevant to the natural history of the species. Thus, experimental treatments should reflect real-world conditions and tease apart the naturally relevant sensory and cognitive capabilities of the organism. Also, behavioural traits that develop gradually, rather than suddenly, provide a greater opportunity to examine quantitative physiological correlates that underlie a particular behaviour and its antecedents. Finally, behaviours that are relatively experience independent have some important advantages from an experimental standpoint: (1) features of the ontogenetic environment, such as the opportunity for social interaction, require less control (e.g., social isolation or deprivation conditions are not necessary) and (2) repeated sampling of behaviour within subjects can often be achieved without influencing subsequent assays (i.e., avoiding test order effects).

Communication is a particularly compelling class of behaviour for developmental studies. As a hallmark of social behaviour, one of its most important functions is to identify individuals as members of a particular group, be it family, friends, sex, or species. Studies of the development of such communication systems are numerous, owing especially to the pioneering studies of song learning in oscines by Thorpe and Marler and the legions of researchers they have spawned [15, 16]. Due to the success of these ventures, however, there is a bias in what we know about the development of communication systems. Most studies have focused on the ontogeny of signal production rather than receiver decoding, and similar emphasis has been given to learning over nonlearning [17–21]. But receivers are a key part of a communication network, and it might be that nonlearned mechanisms are more ubiquitous than learned ones in the communication systems of most animals [22–30].

## 3. What Are the Key Features for the Study of the Evolution of Communication Behaviour?

By exploring the development of nonlearned communication behaviours, we may broaden our understanding of developmental patterns, including the role of constraints in the evolution of communication systems (e.g., evolutionary: phylogenetic inertia, lack of environmental pressure, and pleiotropy; organismal: constraints on memory; environmental: signal transmission [31]). And by considering the potential role of constraints, including physiological processes such as developmental limitations in the plasticity of neuroanatomical connectivity, we might avoid the error of considering each type of behaviour as depending on a distinct mechanism and having a separate adaptive function, a sustained criticism by Schneirla [32, 33]. Instead, seemingly unrelated behaviours may have a common origin. Conversely, assuming that two similar or identical behaviours serve similar functions or share a homologous origin, is another unsatisfactory and potential outcome of ignoring the role of evolutionary constraints. In one simple example that illuminated the problems with this assumption, Beach [34] showed that the lordosis response in neonate guinea pigs is part of an excretory pattern and unrelated functionally and mechanistically to the better-known role for this behaviour in communicating sexual receptivity.

To overcome some of these obstacles, evolutionary studies must examine behaviour in a phylogenetically informed manner. It is not sufficient, for example, to simply compare two or more distantly related species for a character of interest and draw conclusions about the evolutionary forces (or constraints) that gave rise to differences and similarities. This matter cannot be understated and has rightly received recent attention in the context of morphological [35] and behavioural evo-devo [36, 37]. Furthermore, in order to understand the pattern and process of behavioural evo-devo, it is critical that behavioural assays used across taxa are standardized, efficient, and “fair” (i.e., not biased toward a given species [38]). This is often a major challenge and yet another reason to begin by examining behavioural development across a collection of related taxa that share certain life-history characteristics making possible standardized and unbiased tests.

In order to draw evolutionary inferences, it is also important to have a grasp of the natural variation in a group (species, population, sex) and an understanding of the fitness consequences of such variation. Because communication systems evolve as a consequence of selection on both signalers and receivers, it is necessary to consider the implications of ecological selection on both members of a communication dyad.

## 4. What Are the Key Features for the Study of Evo-Devo of Communication Behaviour?

Behavioural evo-devo must satisfy the features essential for both traditional developmental and evolutionary studies of behaviour. This requires a uniquely integrative approach, from gene expression in the brain to ethologically relevant behaviour expressed under naturalistic conditions. For this effort to be tractable, it is essential to begin by examining the evolutionary and developmental basis of behaviours that have limited plasticity. Communication offers such an opportunity, and in fact, is one of the few historical examples of an evo-devo approach applied to behaviour.

The pioneering work in evo-devo by Gilbert Gottlieb was one of the few exceptions to the focus on learned communication in vertebrates. Through a series of seminal studies, Gottlieb established that imprinting on the maternal assembly vocalization in ducklings rests on hearing their own contact vocalizations before they hatch [3]. Gottlieb thus demonstrated that normal species-typical responses to communication signals relied critically on experience (self-stimulated in this instance) despite a lack of vocal learning. As a result of this discovery, the traditional distinction between learned and innate traits began to break down conceptually, and it became clear that “environment” (i.e., “nurture”), as it is traditionally construed, cannot be extracted from genes during ontogeny. This central tenet of evo-devo was perhaps best stated by Hebb [39], when he argued that it is as meaningless to ask how much a given piece of behaviour rests on genes and how much on the environment as it is to ask how much the area of a field depends on its length and how much depends on its width. As a relatively new field, evo-devo attempts to understand the evolutionary and developmental origins of phenotypes, and in doing so, has borrowed a variety of techniques and concepts from, among other disciplines, comparative genetics, cell and molecular biology, physiology, and morphology. What has been largely absent from evo-devo is an emphasis on behaviour (for exceptions, see [3, 40–42]).

Despite early work by Gottlieb, the task of identifying the developmental origins of acoustic communication behaviour has been undertaken largely by studies of vocal and auditory plasticity in birds and humans [8, 29, 43, 44]. As a consequence, our understanding of the ontogeny of communication behaviour in organisms that lack social influences on vocal and auditory learning is limited, despite the fact that learning appears to play little, if any, role in shaping pattern recognition for communication signals in many taxonomic groups, such as insects [10], anuran amphibians [9], nonpasserine birds [8], and many primates [7]. Although it might be assumed that such “hard-wired” behaviour in these groups simply lacks developmental trajectories *per se *(e.g., changes in the frequency of expressing a particular behaviour), this is an untested assumption. Given the widespread pattern of nonlearned communication behaviour in animal taxa, it is all that more important to better grasp the development of such behaviour if we are to understand the evolutionary origins of this class of behaviour.

Behaviour is considered evolutionarily one of the most labile elements of a phenotype [45], and evolutionary change may come about through the adjustment of developmental programs, such as in the beak morphology of Darwin's finches [46], dimorphisms in horn allometries in beetles [47], or the size and shape of the vertebrate brain [48]. We thus argue that it is essential to examine developmental trajectories of both learned and nonlearned behaviours. Toward this end, we review some published studies that consider the developmental origins of species-typical behaviour and the results from recent studies [49, 50] of the developmental origins of species recognition behaviour in the túngara frog (*Physalaemus pustulosus*)—a species that does not exhibit auditory or vocal learning.

In addition to the key features outlined above, we believe the following topics are fundamental lines of inquiry for behavioural evo-devo in the years to come: (1) when do adult behaviours emerge during ontogeny and how do these behaviours and their underlying mechanisms stay in register during development? (2) Do physiological constraints, such as the necessity of establishing, organizing, and maintaining long-distance neuroanatomical connections early in brain development, predispose organisms to express behaviours antecedent to their functional relevance? (3) Do differences in the development of species-specific predispositions provide raw material for sexual selection?

Here we describe a system amenable to these questions. We illustrate the developmental trajectory of behaviour in a species that meets the prerequisites for an evo-devo approach to behaviour and then discuss future comparative studies that would provide insight into these fundamental questions.

### 4.1. Anuran Acoustic Communication and Sexual Behaviour

Species recognition and sexual behaviour are especially relevant classes of behaviour for inquiries into evolutionary development, given their role in maintaining parity between the sexes and the potential for sexual selection to be a driving force in the speciation process [51], as has been suggested specifically in the genus *Physalaemus* (= *Engystomops *[52–54]).

**Figure 1:**
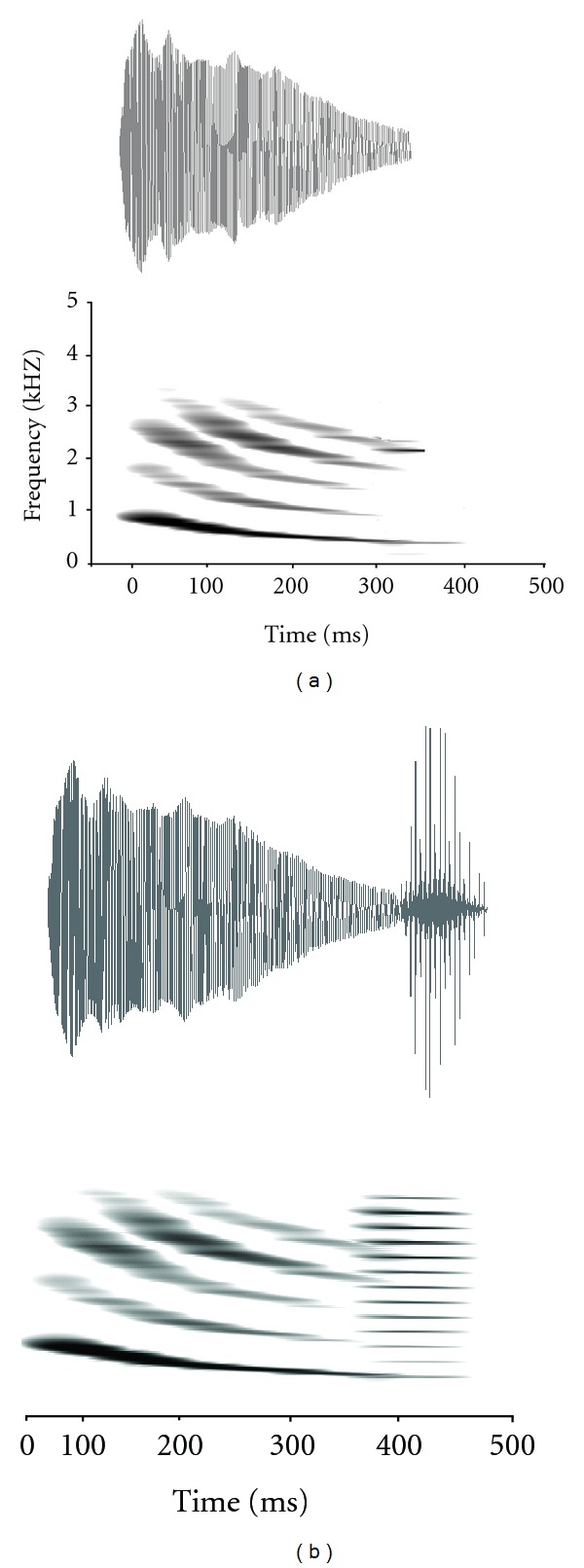
Oscillograms and spectrograms of (a) a natural túngara frog whine and (b) whine-chuck, figure adapted from [49].

As with many animals, communication subserves species recognition and mating behaviour for anuran amphibians. During the breeding season, adult males vocally advertise to reproductive females using species-typical calls—the key trait which females use to localize and select amongst males. In túngara frogs, males produce two types of advertisement signals: first one is simple calls, or “whines”, which are about 300 ms in duration and consist of a harmonically related stack of frequency downsweeps with a dominant frequency beginning at approximately 1000 Hz and terminating at about 400 Hz (Figure 1(a)). Complex calls, or “whine-chucks,” contain the whine component facultatively embellished with 1–7 suffixes, which consist of short (ca. 30 ms) bursts of harmonics with most of their energy concentrated above 1500 Hz (Figure 1(b); reviewed in [55]). Unlike túngara frogs, most members of the genus produce whine-only calls, although there are exceptions [53].

Two auditory end organs in túngara frogs, the amphibian papilla and the basilar papilla, primarily process the whine and chuck components, respectively, based on the frequency tuning of the end organs [56]. Behaviourally, both sexes approach whines and whine-chucks (i.e., exhibit phonotaxis) and evince strong preferences for the whine-chuck; this preference has been the subject of much research in sexual selection [55]. Phonotaxis toward conspecific vocalizations is considered an archetypical sexual behaviour in frogs and toads, and therefore, is expected to represent a key trait for the sexually mature adult phenotype. Phonotaxis is an expression of mate choice in females and presumably serves to direct males to conspecific choruses to join and participate in [49, 57].

### 4.2. Developmental Onset of Species-Typical Phonotaxis

#### 4.2.1. Vocal and Auditory Learning of the Conspecific Advertisement Call Is Absent in Túngara Frogs

Acoustic isolation experiments using male and female túngara frogs demonstrated that males need not hear conspecific signals or interact socially during premetamorphic (larval) and postmetamorphic (juvenile) development in order to produce species-typical advertisement calls as mature adults. Likewise, females isolated acoustically from advertisement signals during development exhibit normal phonotaxis to conspecific signals as reproductive adults [6, 50]. These results confirmed earlier suggestions, based on assumptions of call stereotypy and the consistency of phonotactic preferences, that social experience plays virtually no role in this particular behaviour [9]. The lack of learning during behavioural development, however, has dampened scientific interest in studying the time course for the expression of auditory recognition and discrimination behaviour and physiology in this class of organisms. We believe that this characteristic of the species provides not only logistical advantages mentioned earlier, but also a unique opportunity to determine if and how this nonlearned behaviour develops during growth toward reproductive adulthood.

#### 4.2.2. Conspecific Recognition and Discrimination Is Species Typical in Juvenile Túngara Frogs

Species recognition and sexual behaviour in frogs can be examined by broadcasting the vocal advertisement signal from loudspeakers, thus providing control over the sensory experience and limiting it to this single modality. We performed a series of within-subject phonotaxis experiments on túngara frogs during postmetamorphic development [49]. The results demonstrated that juvenile frogs of both sexes exhibit species recognition and discrimination behaviour with the same selectivity observed in adults. Much like adults, juveniles in two-choice test conditions preferentially approach (1) the normal whine when it was broadcast antiphonally with the temporally reversed version (a frequency upsweep) of the identical call, (2) the normal whine over a synthetic whine intermediate between the conspecific and a heterospecific, and (3) the whine-chuck over the whine.

The key feature of the behaviour that changes during development is the frequency of phonotaxis—both sexes exhibit conspecific phonotaxis in a gradually increasing manner as they approach adulthood over approximately nine months of postmetamorphic development (population response frequency varies from ca. 10% after metamorphosis to ca. 70% at maturity) [49]. While a variety of correlated measures of behaviour follow the same developmental trajectory (e.g., response latency and path length), one component of phonotaxis behaviour emerges in a sharp and sexually differentiated fashion as animals enter reproductive competence: gravid females exhibit the onset of highly localized movement (i.e., “locomotor perseverance”) adjacent to speakers broadcasting conspecific signals, while males, nongravid females, and juveniles do not. The limitation of this behaviour to reproductively active females suggests that locomotor perseverance is related to mate-searching behaviour, while other aspects of phonotaxis behaviour are not specific to mate searching.

These results are relevant to a variety of issues in behavioural evo-devo. First, there has been a suggestion that “premature” forms of species recognition occur in organisms that exhibit vocal or auditory learning of conspecific signals because such traits serve to guide the attention of young animals toward conspecifics to minimize heterospecific learning [28] or function more generally in vertebrates to direct learned perceptual preferences [58]. Given the lack of auditory learning in the túngara frog, however, these results instead suggest that such “premature” traits might signal a more basic constraint of developing vertebrate auditory systems and that the sensory processing circuits containing the necessary feature detectors for conspecific recognition are established before, during or immediately following metamorphosis. Second, despite the potential for considerable morphological, anatomical, and physiological change in the auditory system during postmetamorphic development, the functional selectivity of the system is “in place” and species-typical throughout ontogeny. Also, unlike the birds studied by Gottlieb [3], juvenile frogs do not vocalize, thus eliminating the potential role of vocal self-stimulation in organizing subsequent behavioural preferences. One final point worth noting is that while phonotaxis is considered a sexual behaviour when observed in adults, there remains the possibility that this behaviour serves a nonsexual function in juveniles. While one possible nonsexual function in juveniles can be rejected—that it subserves vocal or auditory learning—there are a host of alternative functions such as conspecific cueing and juvenile dispersal [59, 60]. This point also illustrates the difficulty of determining the relative roles of adaptation and constraint in developing organisms without some reference to the known function of the behaviour in question. Resolving these sources of variation might be achieved through mechanistic studies carried out using a comparative approach, including those outlined in the following sections.

### 4.3. Evo-Devo of Species Recognition in *Physalaemus*

#### 4.3.1. Evo-Devo Prospects in the Genus *Physalaemus*

 The genus *Physalaemus* has a number of potential advantages for use in studies of evolutionary developmental biology. First, the phylogeny in this group is well resolved [52, 61] thus providing the requisite framework for comparative studies. Second, behaviours such as phonotaxis are nonlearned, thus facilitating comparative work as such studies are released from variation due to social interactions in the rearing environment. Lastly, adult sexual behaviour in this genus has been surveyed widely and shows interesting patterns of variation that warrant an evo-devo approach [56, 62–64]. And although developmental studies of behaviour in the genus *Physalaemus* are presently limited to *P. pustulosus*, we know that the adult endpoints across the taxon differ in recognition and discrimination of acoustic advertisement signals, both behaviourally [63, 65] and neurally [56]. Therefore, it follows that these behavioural and physiological differences emerge at some point during development—such species variability during ontogeny is a key prerequisite for evo-devo studies.

#### 4.3.2. Patterns of Immediate-Early Gene Induction during Call Reception in Developing Túngara Frogs

**Figure 2:**
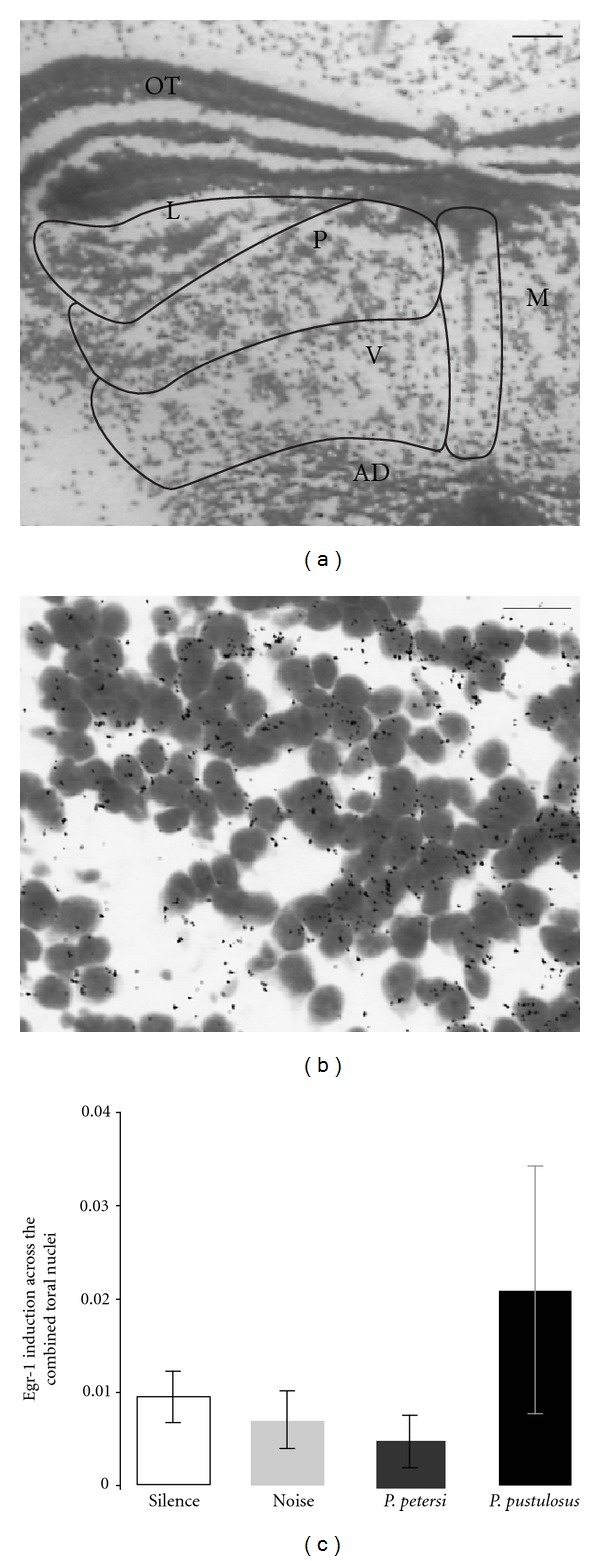
(a) Coronal section of torus semicircularis with subdivision boundaries outlined (L: laminar; P: principal; V: ventral; M: medial; OT: optic tectum; AD: anterodorsal tegmentum). Scale bar 0.1 mm. (b) High magnification showing silver grains over toral cell bodies. Scale bar 0.01 mm. (c) Mean expression levels of egr-1 (silver grains per pixel of cell area ± SEM) from radioactive in situ hybridization combined across four toral subdivisions for each of four acoustic treatments: *P. pustulosus* (*N* = 6), *P. petersi *(*N* = 3), noise (*N* = 2), and silence (*N* = 2). Statistical analyses were not performed due to small sample sizes.

 An aspect of development not accessible to earlier researchers is the ability to identify the ontogeny of neural processing, in this case of recognition signals. Changes in the expression of immediate-early genes (IEGs) such as egr-1 represent the initial genomic response to an inducing stimulus and have been used successfully as markers of neural activation in a variety of species and systems [66]. For example, egr-1 mRNA and protein expression in the auditory system have been an effective measure of regional and functional activation in response to acoustic stimuli in songbirds [67, 68] and adult túngara frogs [69–75]. The anuran auditory midbrain (torus semicircularis) is an excellent candidate for such investigations because it performs sensory processing and sensorimotor integration and has been shown to play a role in regulating acoustically guided behaviour [76]. We performed a preliminary study of IEG induction in juvenile túngara frogs that were three months postmetamorphically using radioactive in situ hybridization in the auditory midbrain. We quantified egr-1 mRNA expression in the brain after exposing juvenile frogs to either conspecific or control stimuli (silence; amplitude modulated and band-pass filtered noise; the heterospecific *P. petersi* mating signal). The results suggest that species typical patterns of activation in the auditory midbrain are established quite early during postmetamorphic development; however, variability in expression patterns appears to be greater in juveniles compared to adults (see Figure 2; for adults see [70]). One possible explanation for the greater variability in activation patterns observed in juveniles compared to adults, particularly in response to the conspecific signal, is that juveniles respond with phonotaxis both less frequently than adults and do so with considerable inter-individual variation. Baugh [77] showed that this individual variation in response probability during development is not the result of a dichtomy (e.g., responders and nonresponders) but rather belongs to a Gaussian distribution of response frequencies suggesting individual differences in the developmental onset of behavioral, and presumably, neural responses. Amphibians are known to exhibit considerable plasticity in the timing developmental events, and we assume that the response properties of the underlying neural circuits involved in processing and responding to auditory signals are likewise affected by the timing of maturational events. Conducting more extensive studies at multiple time points during development could shed light on the mechanistic basis of early species recognition in this frog and thus describe when and how neural and behavioural recognition emerge during development.

The ontogeny of auditory processing is a compelling candidate for comparative studies. IEG studies across members of the *Physalaemus *genus could yield insights into the evolutionary and developmental origins of variation in auditory species recognition. One distinct advantage of the IEG approach is that patterns of activation across most of the brain can be quantified, which enables the simultaneous inspection of low-level sensory processing as well as sensorimotor integration [70, 72]. This application provides for a systems-level approach that allows us to identify candidate neural substrates for species-typical behaviour. Ideally IEG studies will be combined with neuroanatomical and physiological studies that can extend the functional inferences drawn from IEG results. For example, electrophysiological recordings in the laminar nucleus of the auditory midbrain, an area known to integrate both sensory and motor information, could yield information about how whines and chucks are differentially processed and how the response properties of neuronal populations processing chucks compare across members of the genus. Likewise, comparative tract tracing studies that examine the size and distribution of midbrain populations processing acoustic signals that stimulate the basilar papillae (chucks) would inform inferences drawn from functional activation experiments, such as IEG studies. It is critical that developmental IEG studies across species share a set of control stimuli (e.g., silence and synthetic AM noise), so that any intrinsic differences in baseline IEG expression can be accounted for in the analyses.

Complementing IEG studies with behavioural discrimination experiments in a comparative manner (e.g., across members of the *Physalaemus pustulosus* species group) holds significant promise to detail the coordinated neural and behavioural processes undergoing change during development and provide insights into how species differences might arise through differentiated development. Phonotaxis in frogs represents an ideal behavioural assay during development because no animal training is required, and unlike many other species (e.g., songbirds), both sexes perform this behavioural task, thus minimizing the confounding variables of task and sex [57]. It is important to note that phonotaxis is a behaviour exhibited by most anurans and thus provides an unbiased measure to base evolutionary inferences with, an important criterion for evo-devo studies [38]. Moreover, this behaviour can be observed under identical conditions among species using conspecific vocalizations as well as synthetic signals intermediate between selected species pairs, thus providing an excellent method to not only examine when species recognition is present but also when and how subtle forms of species-level discrimination emerge. Further, these approaches could provide insight into the evolutionary developmental basis of female preferences for male traits. By appending chucks to heterospecific whines, Ryan and Rand [65] showed that females of another *Physalaemus* species prefer these novel complex calls, providing support for the theory of sexual selection by sensory exploitation. By replicating these types of behavioural experiments and extending them with mechanistic studies in the brain (e.g., IEGs and cDNA microarrays) during juvenile development across *Physalaemus* species, it might be possible to test hypotheses about how evolutionary changes in developmental programs constrain the universe of possible phenotypes in an evolving lineage.

#### 4.3.3. Transcriptional Profiling in Developing Túngara Frogs

Not only the ontogeny of neural processing of recognition signals could be accessed, but so could the patterns of gene expression that typify the development of these neural pathways. Neural and behavioural analyses in an evo-devo context offer the potential to identify candidate mechanisms of evolutionary change in behaviour, an effort that can be extended to a molecular level using transcriptional profiling across development in multiple species. Transcriptional profiling allows the researcher to compare across contexts (e.g., social stimuli, sex, species, and developmental stage) the levels of expression of large numbers of genes in a given tissue source. Transcriptional profiling can be accomplished using various approaches such as cDNA microarrays or next-generation sequencing methods, with increasing availability for nontraditional genetic model organisms. We propose comparing expression profiles of brain regions that have structural or functional differences that parallel developmental and species differences in behaviour. Focusing research on stages of development prior to and including the emergence of species differences in neural or behavioural traits would provide candidate molecular pathways that may mediate the species specificity (and likewise, species variability) of behaviours. Further analysis of the regulation of these molecular pathways and the differential expression throughout the developing organism using in situ hybridization would generate hypotheses about the potential constraints and pleiotropic effects that may shape the evolution of behaviour. For example, what are the developmental expression patterns for genes regulating the growth and migration of neurons in the auditory system? Do such patterns represent possible developmental constraints on the organization of neuroanatomical connections in auditory-motor pathways that regulate phonotaxis? Do we see species differences in these developmental expression patterns that nominate candidate molecular substrates for sexual selection? Do we find a consistent subset of genes in which expression patterns are more evolutionarily labile across the genus or particular stages of development in which species differences in expression are more pronounced? Given previous research exploring interspecific behavioural variability in this group, classic evo-devo approaches such as these could provide a degree of purchase in understanding the mechanistic bases underlying evolutionary patterns.

#### 4.3.4. Quantitative Genetics in Developing Túngara Frogs

 Quantitative genetics approaches can help link molecular, neural, and behavioural differences between species throughout development to elaborate a detailed view of the evolution of behaviour in an organismal context. The IEG and transcriptional profiling experiments might identify a number of species differences in molecular or neural traits, only some of which are related to the process of species recognition and its evolution. Quantitative genetics studies that incorporate developmental and adult phenotypes at all levels (behaviour, neural, and molecular) can determine the degree to which different sets of phenotypes share genetic architecture. Breeding designs that shuffle genetic material from two or more clades effectively break apart suites of genes that differ among populations or species, revealing which traits cooccur in organisms and therefore might be causally related. Quantitative genetics approaches would therefore enhance our ability to integrate behavioural neural, and molecular phenotypes related to species-typical behaviour and would moreover allow us to analyze possible genetic constraints on evolution, particularly pleiotropic effects on other phenotypes. One prerequisite for the proposed studies is cross-fertility of individuals with very distinct behavior. Although limited information is available about hybrid matings across the genus, at least one interesting cross-breeding experiment is possible. F1 hybrids between two clades of *P. petersi* with highly differentiated male calls and female preferences [53] have been generated and are currently approaching sexual maturity (K. L. Hoke, unpublished).

We can test specific hypotheses such as whether a particular gene expression difference between species in embryonic development is genetically correlated with the mate preferences of adults, thereby narrowing the list of candidate molecular pathways that contribute to particular behavioural phenotypes. Similarly, we can link molecular differences at different stages of development with specific functional neural measures later in life. We can directly assess the degree to which neural and behavioural traits correlate during development and attribute this covariation to genetic or environmental sources. Shared genetic bases for interspecific differences in species recognition behaviours and other phenotypes such as intraspecific mating preferences or body size would expand our view of the constraints and interactions that shape the effects of natural and sexual selection on behaviour.

### 4.4. Developmental Onset of Behaviour in Other Species

 If we extend this inquiry concerning the origins of adult behaviours to other taxa and classes of behavior, there are a small handful of cogent examples of developmental constraints that demonstrate how underlying adult-typical physiology often precedes the onset of particular behaviours. For example, Bentley and Hoy [78] showed that the neural network for generating song in crickets is present during postembryonic development and “in place” before the actual sound-producing structures (the forewings) have developed. This neural network is inhibited until the final moult to adulthood (see also [79]). Likewise, in a study of auditory and vocal ontogeny in the anabantoid fish *Trichopsis vittata*, Wysocki and Ladich [80] demonstrated that, whereas auditory sensitivity in general precedes vocal capabilities, vocalizations precede the capacity for acoustic communication, as juveniles are not sensitive to the dominant frequencies contained in conspecific sounds.

Examples outside of communication behaviour include complex motor patterns such as walking and the righting response in silk moths (*Antheraea pernyi *and *Hyalophora cecropia*). These behaviours are typical of posteclosion adults yet are present in developing pupae and only observed if the pupal cuticle is removed. These behaviours are suppressed shortly before eclosion and then released from inhibition by the eclosion hormone at the final pupation [81]. A related phenomenon was observed in *Antheraea polyphemus*, in which pupae exhibit flight and warm-up motor patterns characteristic of adults in the week preceding pupation [82]. These examples and our studies in frogs point towards ontogenetic precursors to adult behaviour that emerges potentially well before such behaviour is demanded of the organism. The implications for behavioural evo-devo are likely widespread, and these examples barely begin to scratch the surface for what promises to be a compelling direction for future research.

## 5. Conclusions

Species recognition presents an excellent domain for testing hypotheses about the evolutionary developmental origins of behaviour. The integrative studies reviewed and proposed here aim to address both novel and fundamental questions in behavioural evo-devo: (1) when do adult behaviours emerge during ontogeny, and how do these behaviours and their underlying mechanisms stay in register during development? (2) Do physiological constraints, such as the necessity of establishing, organizing, and maintaining long-distance neuroanatomical connections early in brain development, predispose organisms to express behaviours antecedent to their functional relevance? (3) Do differences in the development of species-specific predispositions provide raw material for sexual selection?

To answer these questions requires a system in which developing animals naturally perform, or can be stimulated to exhibit, some form of an adult-typical behaviour. This unique prerequisite is met in *Physalaemus*, which exhibit conspecific phonotaxis early in life. We reviewed studies demonstrating that phonotaxis, widely considered a “sexual” behaviour in anurans, is present in young juveniles of both sexes and is increasingly expressed during growth to adulthood. We argued that whereas the developmental origin of this presumably adult behaviour occurs early, the fully formed version is not present until reproductive onset, suggesting that the principal modification during development is the motivation to respond to conspecific signals in a sexual context. Therefore an investigation of the role that sex steroids play during development (as gonads grow and differentiate) to influence the trajectory of behavioural expression is warranted. An essential area for future research is to determine how these and other aspects of developmental physiology influence the variation available for selection to act on, thus determining the evolutionary trajectory of a lineage by making some phenotypes relatively accessible and others inaccessible [83].
